# Effect of Pilates on Sleep Quality: A Systematic Review and Meta-Analysis of Randomized Controlled Trials

**DOI:** 10.3389/fneur.2020.00158

**Published:** 2020-03-24

**Authors:** Zehua Chen, Xiangling Ye, Zhen Shen, Guoqian Chen, Weijian Chen, Ting He, Xuemeng Xu

**Affiliations:** ^1^The Fifth Clinical Medical School, Guangzhou University of Chinese Medicine, Guangzhou, China; ^2^Kunming Municipal Hospital of Traditional Chinese Medicine, Kunming, China; ^3^Guangzhou Liwan District Orthopedic Hospital, Guangzhou, China; ^4^Guangdong Second Traditional Chinese Medicine Hospital, Guangzhou, China

**Keywords:** sleep, exercise, sleep quality, pilates, Pittsburgh sleep quality index

## Abstract

**Objective:** Pilates exercise is increasingly used to improve sleep quality, but relevant evidence remains unclear. We aimed to estimate the effect of Pilates on sleep quality.

**Methods:** Five databases were searched for articles published until 10 December 2019. Two investigators screened the articles and extracted data from each included study. A meta-analysis was performed to evaluate the effect of Pilates on sleep quality, assessed using the Pittsburgh Sleep Quality Index (PSQI).

**Results:** Six randomized controlled trials (RCTs) comprising 477 participants were included according to the inclusion and exclusion criteria in the study. All included studies reported the positive effects Pilates had on sleep quality. The Pilates group (PG) significantly lowered the PSQI total score (MD = −3.60, 95%CI: [−5.41, −1.78), *P* = 0.0001, *I*^2^ = 97%) compared to the non-exercising control group (CG), whereas no significant improvement in use of sleep medication was observed (MD = −0.33, 95%CI: [−0.73, −0.06), *P* = 0.10, *I*^2^ = 68%). However, in a subgroup analysis, we found that there was no significant reduction in the PSQI total score for healthy participants over 40 years old (MD = −3.73, 95%CI: [−7.89, 0.42], *P* = 0.08, *I*^2^ = 98%) and for postmenopausal women (MD = −5.55, 95%CI: [−13.98, −2.89], *P* = 0.20, *I*^2^ = 98%).

**Conclusions:** Overall, Pilates improved sleep quality but had no significant effect on the use of sleep medication. However, Pilates showed no significant impact on sleep quality for healthy individuals over 40 years old and for postmenopausal women. Well-designed and large-scale RCTs are needed in the future.

## Introduction

Poor sleep is a serious public health problem. It is estimated that the incidence of poor sleep quality was 8.3% among residents (1), and up to 42.5% in the working population (2). Growing evidence suggests that it is also quite prevalent in the middle-aged population (3), postmenopausal women (4), the elderly (5), cancer patients (6), and those suffering from mental and chronic diseases (3). Even worse, as is reported, it serves as a risk factor for depression and hypertension (7), neurodegenerative disorders (8), and cardiovascular diseases (9), and it even increases mortality (10), which heavily compromises people's health and imposes a constant and growing economic burden on patients and society. It is therefore urgent and crucial to explore a safe, effective, economic, and feasible method to address sleep problems. Until now, many therapeutic methods have been developed, of which, exercise has been advocated for treating insomnia because it has very few side effects (5). Recent meta-analysis studies (11, 12) suggest a potential benefit of program/mind-body exercise to improve sleep quality in clinic patients and healthy individuals.

Pilates exercise originated in the 1920's, targeting the body's core muscles (13). As a form of mind-body exercise, the Pilates exercise system mixes practical movement styles and ideas of gymnastics, martial arts, yoga, and dance with philosophical notions, which are based on six fundamental principles: concentration, control, centering, flowing movements, precision, and breathing (14). Different from yoga, Pilates pays more attention to awareness, breathing, and core muscles. For one thing, Pilates exercise has been viewed as a potential strategy for the rehabilitation and adjuvant treatment of chronic diseases in the clinic (15–17). Furthermore, it also has been widely applied in health subjects because of its positive effects (18, 19). As reported by Leopoldino et al. (20), Pilates can significantly improve sleep quality. However, this improvement was not significantly detected in another observational study (21). Thus, clinical data that examines the effect of Pilates on sleep quality remains controversial. Consequently, we carried out a systematic review and meta-analysis of randomized controlled trials (RCTs) to evaluate the effect of Pilates on sleep quality and further provide reference for clinical practice.

## Methods

This meta-analysis was fulfilled according to the Preferred Reporting Items for Systematic Review and Meta-analyses (22).

### Selection Criteria

All the literatures were considered to be eligible if they met the following criteria: ① Study design: clinical randomized controlled study; ② Population: without restrictions; ③ Intervention: Pilates, Pilates with other therapies; ④ Outcomes: at least one efficacy index related to sleep quality; ⑤ Comparisons: Pilates vs. other therapies, Pilates with other therapies vs. other therapies, Pilates vs. no intervention; ⑥ Language: English and Chinese. A literature would be excluded if it met any of the following criteria: ① Non-RCTs, reviews, full-text unavailable articles, case reports; ② Repeated publications, animal experimental studies.

### Search Strategy

We conducted the literature search in PubMed, EMBASE, Web of science, CINAHL, and the China National Knowledge Infrastructure (CNKI) database. Keywords such as “Pilates,” “Pilates-based exercise,” “randomized controlled trial,” “exercises, Pilates-based,” “Pilates Based Exercises,” “Pilates Training” and “Training, Pilates” were used to search without restrictions, from inception to 10 December 2019. The search strategy is shown in Supplementary eFigure 1. Two independent researchers (ZC and XY) screened all the literature used in this study. First, all literature was preliminarily selected by reading the titles and abstracts after duplicates were removed. Second, the full text of the remaining articles was read carefully and screened strictly based on the inclusion and exclusion criteria. Finally, we extracted the data and materials in the literature included, while discrepancies between the two reviewers were solved by consulting a third reviewer and discussing until a consensus was reached.

### Data Extraction

The main information used in our study was collected from the included articles. This included author names, region, publication year, age, study design, intervention type, intervention dose, and the main outcomes.

### Risk of Bias and Quality Assessment

Two investigators (ZC and WC) assessed the Cochrane Collaboration risk of bias table (23) and the quality of the literature using the Jadad score scale (24). We also examined the public bias using Begg's test and Egger's test (25).

### Statistical Analysis

We performed the data analysis using review manager 5.3 software for the literature included, and the results were intuitively illustrated by the forest map. The Begg's and Egger's tests were evaluated using Stata 14 (USA, StataCorp LP, 2015). In this study, all parameters were continuous variables. They were pooled by mean differences (MDs) with 95% confidence intervals (95% CI). Heterogeneity was assessed by the Cochran Q-test and *I*^2^ index (26). If heterogeneity was not significant (*I*^2^ < 50%), fixed effect models were used; random effect models were applied when heterogeneity was significant (*I*^2^ ≥ 50%). The difference was statistically significant when *P* < 0.05.

## Results

### Study Selection

**Figure 1:**
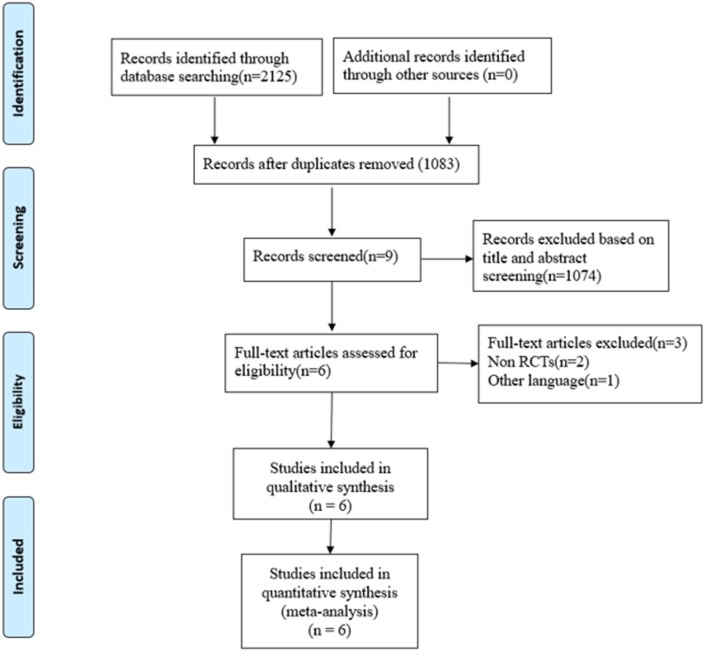
Flowchart of study selection.

A total of 2,125 related records were identified by searching five databases. All the literature was imported into EndNote X8 (Bld, 10063) to remove duplicates. After removing 1,042 duplicates and eliminating 1,077 articles through a strict step by step screening, six trials (27–32) comprising 477 participants were reviewed. Three studies were excluded: a poster, a protocol, and one in Farsi. All included studies recorded a positive influence of Pilates on sleep quality, in which the Pittsburgh Sleep Quality Index (PSQI) (33) was the only and the same evaluation index for sleep quality. The selection flowchart is shown in Figure 1, and the characteristics of each included study are summarized in Table 1.

**Table 1:** Characteristics of the included studies.

**References**	**Country**	**Age (years)**		**Sample size**	**Population group**	**Intervention**		**Study design**	**Intervention dose**	**Main outcome**	**Jadad**
		**PG**	**CG**	**(PG/CG)**		**PG**	**CG**				
Ahmadinezhad et al. (27)	Iran	54.08 ± 3.84		36/36	Postmenopausal women	PE	WI	RCT	3 sessions/w,6w	abcdefgh	3
Aibar-Almazán et al. (28)	Spain	66.79 ± 10.14	69.98 ± 7.83	52/55	Postmenopausal women	PE	WI	RCT	2 sessions/w,12w	abcdefgh	5
Ashrafinia et al. (29)	Iran	24.4 ± 3.6	24.6 ± 3.6	40/40	Postpartum women	PE	Education	RCT	5 sessions/w,5w	abcdefh	2
Curi et al. (30)	Brazil	64.25 ± 0.14	63.75 ± 0.08	31/30	Elderly women	PE	WI	RCT	2 sessions/w,16w	abcdefgh	3
Garcia-Soidan et al. (31)	Spain	47.6 ± 0.8	47.7 ± 0.8	48/51	Middle-aged people	PE	WI	RCT	2 sessions/w,3M	aijk	2
Yang et al. (32)	China	51.24 ± 11.24	51.03 ± 10.01	29/29	Hemodialysis patients	PE+RC	RC	RCT	2~3 sessions/w,6M	a	2

Group: PG, Pilates group; CG, control group; Intervention: PE, Pilates exercise; WI, without intervention; RC, routine care; Study design: RCT, randomized controlled trials.

*Main outcome: a: PSQI (Pittsburgh sleep quality index) total score; b: subjective sleep quality; c: sleep latency; d: sleep duration; e: sleep efficiency; f: sleep disturbances; g: use of sleeping medication; h: daytime dysfunction*.

### Risk of Bias and Quality Assessment

**Figure 2:**
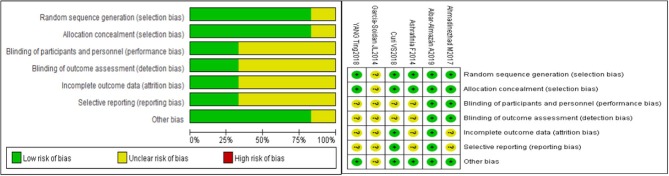
Risk of bias graph.

Risk-of-bias assessment is shown in Figure 2. All studies were described as a random generation, and five articles (27–30, 32) described the methods of randomization in detail. The blind method was detailed in two studies, one double-blinded trial (28) and one single-blinded trial (27). The drop-out rate was recorded in two articles (28, 30). The average Jadad score of the six included studies was 2.67, which indicated that the quality of the studies was fair to mild.

### Meta-Analyses

**Figure 3:**
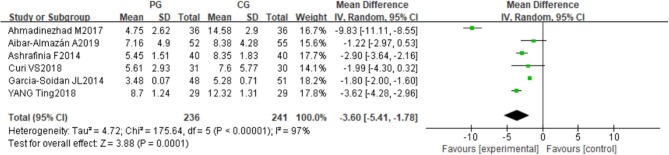
Meta-analysis and forest plot and for PSQI.

All the included studies reported the PSQI. It comprises nine questions evaluating, seven aspects including subjective sleep quality, sleep latency, sleep duration, sleep efficiency, sleep disturbances, use of sleeping medication, and daytime dysfunction. A significant difference was observed between the Pilates group (PG) and the control group (CG) in the PSQI total scores (MD = −3.60, 95%CI: [−5.41, −1.78], *P* = 0.0001, *I*^2^ = 97%) (Figure 3). The PG decreased the scores when compared to the CG in subjective sleep quality (MD = −0.71, 95%CI: [−1.35, −0.06], *P* = 0.03, *I*^2^ = 96%), sleep latency (MD = −0.57, 95%CI: [−0.73, −0.41], *P* < 0.00001, *I*^2^ = 94%); sleep duration (MD = −0.65, 95%CI: [−1.25, −0.05], *P* = 0.03, *I*^2^ = 94%), sleep efficiency (MD = −0.78, 95%CI: [−0.99, −0.57], *P* < 0.00001, *I*^2^ = 93%); sleep disturbances (MD = −0.15, 95%CI: [−0.25, −0.04], *P* =0.007, *I*^2^ = 79%); and daytime dysfunction (MD = −0.81, 95%CI: [−0.93, −0.68], *P* < 0.00001, *I*^2^ = 97%). However, there was no statistically significant difference between the PG and CG in the use of sleep medication (MD = −0.33, 95%CI: [−0.73, −0.06], *P* = 0.10, *I*^2^ = 68%) (Supplementary eFigure 2).

### Sensitivity and Subgroup Analyses

In this study, the heterogeneity of effects detected among studies was moderate to high. We conducted the sensitivity analysis for the PSQI total score by removing studies from the analysis individually, but the overall heterogeneities and results did not substantially change (Table 2). The subgroup analysis consisted of participants' ages and health conditions. Compared to the CG, we found that there was no significant reduction in the PSQI score for healthy participants over 40 years old, reported in four studies (27.28.29.30) (MD = −3.73, 95%CI: [−7.89, 0.42], *P* = 0.08, *I*^2^ = 98%), and postmenopausal women reported in two studies (27.28) (MD = −5.55, 95%CI: [−13.98, −2.89], *P* = 0.20, *I*^2^ = 98%) (Supplementary eFigure 3).

**Table 2:** Sensitivity analysis for PSQI.

**References**	**Effect size**	**95%CI**	***P***	***I*^**2**^**
Ahmadinezhad et al. (27)	−2.43	−3.42, −1.45	*P* < 0.00001	88%
Aibar-Almazán et al. (28)	−4.04	−6.07, −2.00	*P* = 0.0001	98%
Ashrafinia et al. (29)	−3.73	−6.12, −1.34	*P* = 0.002	98%
Curi et al. (30)	−3.86	−5.86, −1.86	*P* = 0.0002	98%
Garcia-Soidan et al. (31)	−3.97	−6.41, −1.53	*P* = 0.001	96%
Yang et al. (32)	−3.58	−6.00, −1.16	*P* = 0.004	97%

*CI, confidence intervals*.

### Publication Bias

We found that publication bias was negative when evaluated using Egger's regression (*P* = 0.057) and Begg's tests (*P* = 0.06).

## Discussion

To the best of our knowledge, this is the first systematic review and meta-analysis to evaluate the effectiveness of Pilates exercise in sleep quality. In our study, Pilates exercise was found to significantly improve sleep quality on the whole, whereas no significant improvement in use of sleep medication was observed. However, of note, Pilates is not that effective in changing sleep quality in healthy individuals over 40 years old and postmenopausal women.

Sleep plays an important role in health and longevity (34). Because of the prevalence of poor sleep quality, more and more problems related to sleep disorders including fatigue, depression, obesity, and cardiac sudden death are on the rise. Thus, increasing attention has been paid to sleep problems and a growing number of medical staff have focused on economically feasible and applicable therapies for improving sleep quality. As a core concept in sleep, sleep quality is largely subjective, so it is always evaluated using PSQI, a subjective self-report questionnaire, which is considered an accurate and effective method in clinical care and research, and is considered to be cheaper and more readily available than the gold standard measurement of sleep characteristics-polysomnography (35). Due to very few side effects, exercise, as a non-pharmaceutical and relatively safe activity, is widely chosen in clinical practice (36). It is reported that exercise can improve body composition and fitness, which is critical in enhancing sleep quality (37). As for Pilates, it was reported that beneficial effects on sleep quality assessed with the PSQI were recorded in the included RCTs. Newton et al. (38) reported that Pilates, similar to yoga, could obviously reduce the PSQI score for women suffering from sleep disorders when compared to usual activity. On the contrary, Buchanan et al. (39) suggested that there was no significant sleep quality improvement for postmenopausal women. In the present study, overall, Pilates was associated with a significant reduction in PSQI total score by −3.60 (*P* = 0.0001). Interestingly, an important finding that was observed from our subgroup analysis was that PSQI scores did not significantly reduce in PG compared to CG for healthy individuals over 40 years old and postmenopausal women (*P* > 0.05).

Pilates exercise was invented and developed by Joseph Pilates, which emphasized strengthening of the core muscles, increasing flexibility, and enhancing breathing (40). As a mind-body exercise, previous meta-analysis studies (41–44) indicate that the effects of Pilates exercise is mainly reflected in improving mental health, pain, flexibility, fitness, balance, and physical function. Moreover, some studies suggest that Pilates exert positive effects on body satisfaction, attitude, and quality of life (45), and consequently improves health (13), which is helpful for sleep quality. However, sleep regulation is very complex and involves many factors (46). In postmenopausal and postpartum women and the elderly, the factors that affect sleep quality are particularly varied and complex. As reported, sleep problems result from hormone changes, mood disorders, vasomotor symptoms and so on in postmenopausal women (47). In addition, Bei et al. (48) reported that stress, infant behavior, and nighttime labor affect sleep quality among postpartum women. In contrast, in the elderly, it is the chronic medical condition that serves as the main cause impairing sleep quality (49). Accordingly, sleep quality varies among different populations affected by different factors. In our meta-analysis Pilates exercise possesses positive overall effects on sleep quality, but the benefit in sleep quality for healthy individuals over 40 years old and postmenopausal women was not significant because of so many different factors. Therefore, it is reported that exercises need to be further evaluated to judge their usefulness in the treatment of people suffering from sleep problems (50). Meanwhile, different demographic characteristics of the participants in the six included studies may also contribute to heterogeneity.

There are some important limitations in this review. First, a small number of studies were included. Second, random and blind methods of the included studies were rarely provided in detail. Third, Pilates procedure processes, time of duration, and dosages varied from one study to another without a consolidated standard, which can be a potential source of clinical heterogeneity that affects the results. Fourth, this study was conducted at a study level and it is difficult to address or incorporate individual factors at the patient level.

## Conclusion

In this analysis, we systematically reviewed and quantified the effect of Pilates on sleep quality. Overall, Pilates improved sleep quality but had no significant effect on the use of sleeping medication. However, for healthy individuals over 40 years old and postmenopausal women, Pilates showed no satisfactory results and the positive effect was not significant. Given the limitations of this work, additional well-designed and large-scale RCTs and systemic reviews are needed to confirm these findings in the future.

## Data Availability Statement

The datasets analyzed in this article are not publicly available. Requests to access the datasets should be directed to 630327511@qq.com.

## Author Contributions

ZC and XX designed the study. ZC and ZS performed the literature searches and designed the data-extraction form. ZC and XY selected the studies. ZC and WC extracted the data. GC and ZS performed the statistical analyses. XX supervised the study. TH did the language editing. All authors read and approved the submitted version.

### Conflict of Interest

The authors declare that the research was conducted in the absence of any commercial or financial relationships that could be construed as a potential conflict of interest.

## References

[1] Wu W Jiang Y Wang N Zhu M Liu X Jiang F. Sleep quality of Shanghai residents: population-based cross-sectional study. Qual Life Res. (2019). 10.1007/s11136-019-02371-x. [Epub ahead of print]. 31782018

[2] Visvalingam N Sathish T Soljak M Chua AP Dunleavy G Divakar U. Prevalence of and factors associated with poor sleep quality and short sleep in a working population in Singapore. Sleep Health. (2019). 10.1016/j.sleh.2019.10.008. [Epub ahead of print]. 31836498

[3] M.M. Ohayon. Epidemiology of insomnia: what we know and what we still need to learn Sleep Med. Rev. (2002) 6:97–111. 10.1053/smrv.2002.0186 12531146

[4] Kalmbach DA Cheng P Arnedt JT Cuamatzi-Castelan A Atkinson RL Fellman-Couture C. Improving daytime functioning, work performance, and quality of life in postmenopausal women with insomnia: comparing cognitive behavioral therapy for insomnia, sleep restriction therapy, and sleep hygiene education. J Clin Sleep Med. (2019) 15:999–1010. 10.5664/jcsm.7882 31383238 PMC6622507

[5] Moreno Reyes P Muñoz Gutiérrez C Pizarro Mena R Jiménez Torres S Effects of physical exercise on sleep quality, insomnia, and daytime sleepiness in the elderly. A literature review. Rev Esp Geriatr Gerontol. (2020) 51:42–9. 10.1016/j.regg.2019.07.003. 31610889

[6] Roscoe JA Kaufman ME Matteson-Rusby SE Palesh OG Ryan JL Kohli S. Cancer-related fatigue and sleep disorders. Oncologist. (2007) 12(Suppl.1):35–42. 10.1634/theoncologist.12-S1-35 17573454

[7] Kawada T. Risk factors of insomnia in the elderly with special reference to depression and hypertension. Psychogeriatrics. (2019). 10.1111/psyg.12492. [Epub ahead of print]. 31797494

[8] Shamim SA Warriach ZI Tariq MA Rana KF Malik BH. Insomnia: risk factor for neurodegenerative diseases. Cureus. (2019) 11:e6004. 10.7759/cureus.6004 31807391 PMC6876903

[9] Zheng B Yu C Lv J Guo Y Bian Z Zhou M Insomnia symptoms and risk of cardiovascular diseases among 0.5 million adults: a 10-years cohort. Neurology. (2019) 93:e2110–20. 10.1212/WNL.0000000000008581 31694922 PMC6937485

[10] Kabat GC Xue X Kamensky V Zaslavsky O Stone KL Johnson KC. The association of sleep duration and quality with all-cause and cause-specific mortality in the women's health initiative. Sleep Med. (2018) 50:48–54. 10.1016/j.sleep.2018.05.015 29982090

[11] Rubio-Arias JÁ Marín-Cascales E Ramos-Campo DJ Hernandez AV Pérez-López FR. Effect of exercise on sleep quality and insomnia in middle-aged women: a systematic review and meta-analysis of randomized controlled trials. Maturitas. (2017) 100:49–56. 10.1016/j.maturitas.2017.04.003 28539176

[12] Wang X Li P Pan C Dai L Wu Y Deng Y. The effect of mind-body therapies on insomnia: a systematic review and meta-analysis. Evid Based Complement Alternatb Med. (2019) 2019:9359807. 10.1155/2019/9359807 30894878 PMC6393899

[13] Shand D. Pilates to pit. Lancet. (2004) 363:1340. 10.1016/S0140-6736(04)16085-6 15110489

[14] Latey P The Pilates method: history and philosophy. J Bodyw Mov Ther. (2001) 5:275–82. 10.1054/jbmt.2001.0237

[15] Eliks M Zgorzalewicz-Stachowiak M Zenczak-Praga K. Application of Pilates-based exercises in the treatment of chronic non-specific low back pain: state of the art. Postgrad Med J. (2019) 95:41–5. 10.1136/postgradmedj-2018-135920 30636192 PMC6581086

[16] Gonzáles AI Nery T Fragnani SG Pereira F Lemos RR Bezerra PP. Pilates exercise for hypertensive patients: a review of the literature. Altern Ther Health Med. (2016) 22:38–43. 27622959

[17] Fernández-Rodríguez R Álvarez-Bueno C Ferri-Morales A Torres-Costoso AI Cavero-Redondo I Martínez-Vizcaíno V. Pilates method improves cardiorespiratory fitness: a systematic review and meta-analysis. J Clin Med. (2019) 8:E1761. 10.3390/jcm8111761 31652806 PMC6912807

[18] Hornsby E Johnston LM. Effect of pilates intervention on physical function of children and youth: a systematic review. Arch Phys Med Rehabil. (2020) 101:317–28. 10.1016/j.apmr.2019.05.023 31152703

[19] Bueno de Souza RO Marcon LF Arruda ASF Pontes Junior FL Melo RC. Effects of mat pilates on physical functional performance of older adults: a meta-analysis of randomized controlled trials. Am J Phys Med Rehabil. (2018) 97:414–25. 10.1097/PHM.0000000000000883 29283899

[20] Leopoldino AA Avelar NC Passos GB Jr Santana NÁ Jr Teixeira VP Jr de Lima VP. Effect of Pilates on sleep quality and quality of life of sedentary population. J Bodyw Mov Ther. (2013) 17:5–10. 10.1016/j.jbmt.2012.10.001 23294677

[21] Caldwell K Harrison M Adams M Triplett NT. Effect of Pilates and taiji quan training on self-efficacy, sleep quality, mood, and physical performance of college students. J Bodyw Mov Ther. (2009) 13:155–63. 10.1016/j.jbmt.2007.12.001 19329051

[22] Moher D Liberati A Tetzlaff J Altman DG PRISMA Group. Preferred reporting items for systematic reviews and meta-analyses: the PRISMA statement. Int J Surg. (2010) 8:336–41. 10.1016/j.ijsu.2010.02.007 20171303

[23] Higgins JP Altman DG Gotzscbe PC Jüni P Moher D Oxman AD The Cochrane Collaboration' s tool for assessing risk of bias in randomised trials. BMJ. (2011) 343:889–93. 10.1136/bmj.d5928 PMC3196245 22008217

[24] Jadad AR Moore RA Carroll D Jenkinson C Reynolds DJ Gavaghan DJ. Assessing the quality of reports of randomized clinical trials: is blinding necessary? Control Clin Trials. (1996) 17:1–12. 10.1016/0197-2456(95)00134-4 8721797

[25] Shen Z Zheng S Chen G Li D Jiang Z Li Y. Efficacy and safety of platelet-rich plasma in treating cutaneous ulceration: a meta-analysis of randomized controlled trials. J Cosmet Dermatol. (2019) 18:495–507. 10.1111/jocd.12853 30912259

[26] Huedo-Medina TB Sánchez-Meca J Marín-Martínez F Botella J. Assessing heterogeneity in meta-analysis: Q statistic or I^2^ index? Psychol Methods. (2006) 11:193–206. 10.1037/1082-989X.11.2.193 16784338

[27] Ahmadinezhad M Kargar M Vizeshfar F Hadianfard MJ. Comparison of the effect of acupressure and pilates-based exercises on sleep quality of postmenopausal women: a Randomized controlled trial. Iran J Nurs Midwifery Res. (2017) 22:140–6. 10.4103/1735-9066.205954 28584553 PMC5442996

[28] Aibar-Almazán A Hita-Contreras F Cruz-Díaz D de la Torre-Cruz M Jiménez-García JD Martínez-Amat A. Effects of Pilates training on sleep quality, anxiety, depression and fatigue in postmenopausal women: a randomized controlled trial. Maturitas. (2019) 124:62–7. 10.1016/j.maturitas.2019.03.019 31097181

[29] Ashrafinia F Mirmohammadali M Rajabi H Kazemnejad A Sadeghniiathaghighi K Amelvalizadeh M. The effects of Pilates exercise on sleep quality in postpartum women. J Bodyw Mov Ther. (2014) 18:190–9. 10.1016/j.jbmt.2013.09.007 24725785

[30] Curi VS Vilaça J Haas AN Fernandes HM. Effects of 16-weeks of Pilates on health perception and sleep quality among elderly women. Arch Gerontol Geriatr. (2018) 74:118–22. 10.1016/j.archger.2017.10.012 29096225

[31] Garcia-Soidan JL Giraldez VA Zagalaz JC Lara-Sánchez AJ. Does pilates exercise increase physical activity, quality of life, latency, and sleep quantity in middle-aged people? Percept Mot Skills. (2014) 119:838–50. 10.2466/29.25.PMS.119c30z9 25456245

[32] Yang T Shen M Tang XM Xu YF Wan QM Deng LH Effects of Pilates exercise on fatigue and sleep quality in maintenance hemodialysis patients. Chin J Blood Purif . (2018) 17:456–60.

[33] Buysse D J Reynolds C F III Monk T H Berman SR Kupfer DJ. The pittsburgh sleep quality index: a new instrument for psychiatric practice and research. Psychiatry Res. (1989) 28:193–213. 10.1016/0165-1781(89)90047-4 2748771

[34] Grandner MA. Sleep, health, and society. Sleep Med Clin. (2017) 12:1–22. 10.1016/j.jsmc.2016.10.012 28159089 PMC6203594

[35] Seidi PAM Mohammadi H Khazaie H Abas NQ Jaff D. Psychometric properties of the Kurdish version of pittsburgh sleep quality index. Sleep Med. (2019) 63:75–81. 10.1016/j.sleep.2019.04.022 31606652

[36] Kelley GA Kelley KS. Exercise and sleep: a systematic review of previous meta-analyses. J Evid Based Med. (2017) 10:26–36. 10.1111/jebm.12236 28276627 PMC5527334

[37] Dolezal BA Neufeld EV Boland DM Martin JL Cooper CB Interrelationship between sleep and exercise: a systematic review. Adv Prev Med. (2017) 2017:1364387 10.1155/2017/1364387 28458924 PMC5385214

[38] Newton KM Reed SD Guthrie KA Sherman KJ Booth-LaForce C Caan B. Efficacy of yoga for vasomotor symptoms: a randomized controlled trial. Menopause. (2019) 21:339–346. 10.1097/GME.0b013e31829e4baa 24045673 PMC3871975

[39] Buchanan DT Landis CA Hohensee C Guthrie KA Otte JL Paudel M. Effects of yoga and aerobic exercise on actigraphic sleep parameters in menopausal women with hot flashes. J Clin Sleep Med. (2017) 13:11–8. 10.5664/jcsm.6376 27707450 PMC5181601

[40] Joyce AA Kotler DH. Core training in low back disorders: role of the pilates method. Curr Sports Med Rep. (2017) 16:156–61. 10.1249/JSR.0000000000000365 28498224

[41] Fleming KM Herring MP. The effects of pilates on mental health outcomes: a meta-analysis of controlled trials. Complement Ther Med. (2018) 37:80–95. 10.1016/j.ctim.2018.02.003 29609943

[42] Espíndula RC Nadas GB Rosa MID Foster C Araújo FC Grande AJ. Pilates for breast cancer: a systematic review and meta-analysis. Rev Assoc Med Bras. (1992) (2017) 63:1006-12. 10.1590/1806-9282.63.11.1006 29451666

[43] Moreno-Segura N Igual-Camacho C Ballester-Gil Y Blasco-Igual MC Blasco JM. The effects of the pilates training method on balance and falls of older adults: a systematic review and meta-analysis of randomized controlled trials. J Aging Phys Act. (2018) 26:327–44. 10.1123/japa.2017-0078 28771109

[44] Miranda S Marques A. Pilates in noncommunicable diseases: a systematic review of its effects. Complement Ther Med. (2018) 39:114–30. 10.1016/j.ctim.2018.05.018 30012382

[45] Roh SY. The effects of body image, commitment, and attitude on behavior after purchase of Pilates consumers. J Exerc Rehabil. (2018) 14:944–53. 10.12965/jer.1836436.218 30656153 PMC6323345

[46] Murillo-Rodriguez E Arias-Carrion O Zavala-Garcia A Sarro-Ramirez A Huitron-Resendiz S Arankowsky-Sandoval G. Basic sleep mechanisms: an integrative review. Cent Nerv Syst Agents Med Chem. (2012) 12:38–54. 10.2174/187152412800229107 22524274

[47] Lee J Han Y Cho HH Kim MR. Sleep disorders and menopause. J Menopausal Med. (2019) 25:83–7. 10.6118/jmm.19192 31497577 PMC6718648

[48] Bei B Coo S Trinder J. Sleep and mood during pregnancy and the postpartum period. Sleep Med Clin. (2015) 10:25–33. 10.1016/j.jsmc.2014.11.011 26055670

[49] Feinsilver SH Hernandez AB. Sleep in the elderly: unanswered questions. Clin Geriatr Med. (2017) 33:579–96. 10.1016/j.cger.2017.06.009 28991652

[50] Riemann D Baglioni C Bassetti C Dolenc Groselj L Ellis JG Espie CA. European guideline for the diagnosis and treatment of insomnia. J Sleep Res. (2017) 26:675–700. 10.1111/jsr.12594 28875581

