# Supplementary material for: Effect of Pilates on Sleep Quality: A Systematic Review and Meta-Analysis of Randomized Controlled Trials
Source: Front Neurol. 2020 Mar 24;11:158. doi: 10.3389/fneur.2020.00158 (PMC7105773; doi:10.3389/fneur.2020.00158)
Supplement: Supplementary file 1 [file Table_1.DOCX]

Supplementary Material

The search strategy used for Pubmed:

#1. ((((((Pilates-Based Exercises) OR Exercises, Pilates-Based) OR Pilates Based Exercises) OR Pilates Training) OR Training, Pilates)) OR pilates

#2. (((randomized controlled trial[Publication Type] OR randomized[Title/Abstract] OR placebo[Title/Abstract])))

#3. #1 AND #2

The search strategy used for EMBASE:

#1 Pilates-Based Exercises

#2 Exercises, Pilates-Based

#3 Pilates Based Exercises

#4 Pilates Training

#5 Training, Pilates

#6 Pilates

#7 'randomized controlled trial'/exp

#8 #1 OR #2 OR #3 OR #4 OR #5 OR #6

#9 #7 AND #8

The search strategy used for CINAHL:

#1 (Pilates-Based Exercises):ti,ab,kw (Word variations have been searched)

#2 (Exercises, Pilates-Based):ti,ab,kw (Word variations have been searched)

#3 (Pilates Based Exercises):ti,ab,kw (Word variations have been searched)

#4 (Pilates Training):ti,ab,kw (Word variations have been searched)

#5 (Training, Pilates):ti,ab,kw (Word variations have been searched)

#6 (Pilates):ti,ab,kw (Word variations have been searched)

#7 #1 OR #2 OR #3 OR #4 OR #5 OR #6

The search strategy used for Web of science:

TS=(Pilates-Based Exercises OR Exercises, Pilates-Based OR Pilates Based Exercises OR Pilates Training OR Training, Pilates OR Pilates) AND TS=(randomized controlled trial OR randomized)

**CNKI:**

主题=普拉提 or 主题= Pilates or ( 题名= 普拉提 or 题名= Pilates) (精确匹配)

**Supplementary eFigure 1** The search strategy in this review.

a
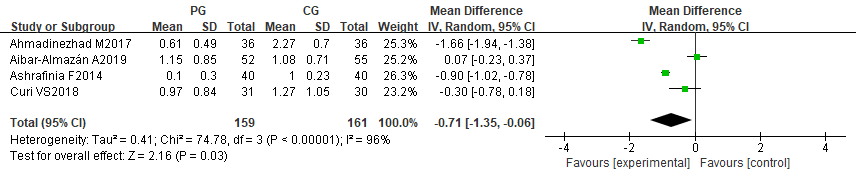


b
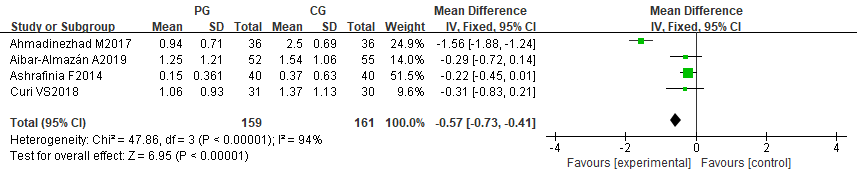


c
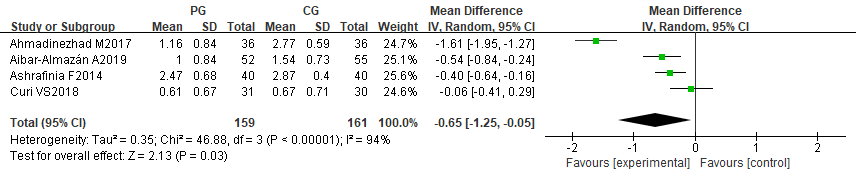


d
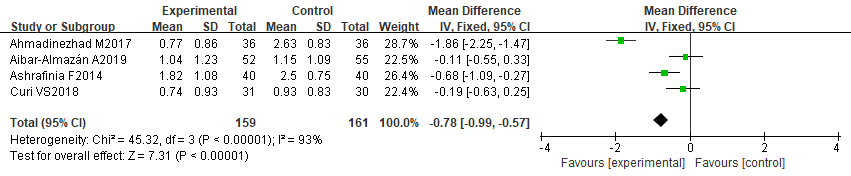


e
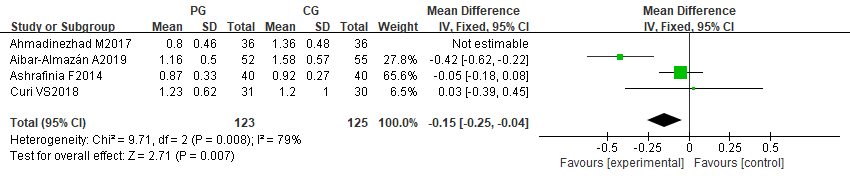


f
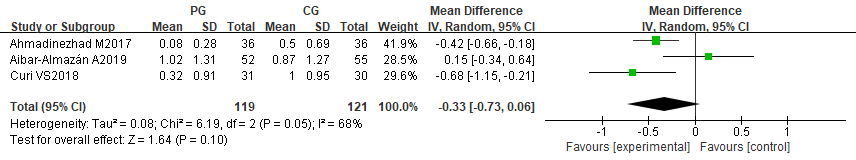


g
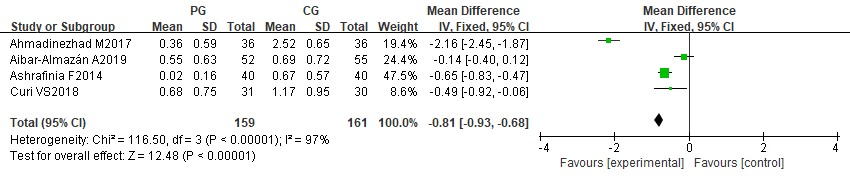


**Supplementary eFigure 2** Meta‐analysis and forest plot and for seven components of PSQI.

PSQI : Pittsburgh Sleep Quality Index; a: subjective sleep quality; b: sleep latency; c: sleep duration; d: sleep efficiency; e: sleep disturbances; f: use of sleeping medication; g: daytime dysfunction


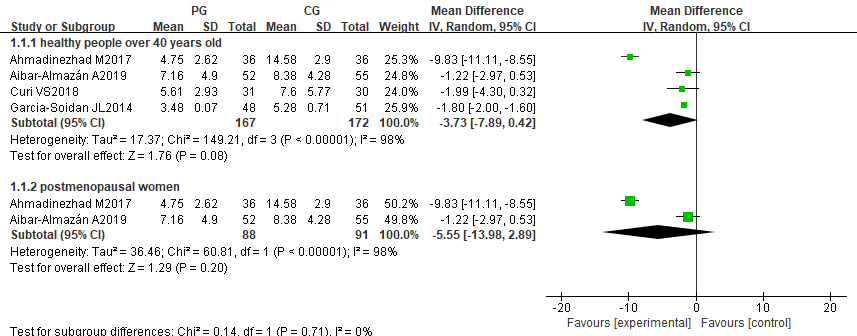


**Supplementary eFigure 3** Subgroup analyses.
